# Insulin Resistance as Related to Psychiatric Disorders in Obese Children

**DOI:** 10.4274/jcrpe.0055

**Published:** 2018-11-29

**Authors:** Deniz Özalp Kızılay, Şermin Yalın Sapmaz, Semra Şen, Yekta Özkan, Betül Ersoy

**Affiliations:** 1 Çiğli State Training Hospital, Clinic of Pediatrics, Division of Pediatric Endocrinology, İzmir, Turkey; 2 Manisa Celal Bayar University Faculty of Medicine, Department of Child and Adolescent Psychiatry, Manisa, Turkey; 3 Manisa Celal Bayar University Faculty of Medicine, Department of Pediatrics, Division of Pediatric Infectious Disease, Manisa, Turkey; 4 Manisa Celal Bayar University Faculty of Medicine, Department of Pediatrics, Division of Pediatric Endocrinology and Metabolism, Manisa, Turkey

**Keywords:** Child, obesity, insulin resistance, mental disorder

## Abstract

**Objective:**

The current study aimed to investigate psychiatric consequences of obesity and the relationship between componenets of the metabolic syndrome and psychiatric disorders in children. Our second aim was to elucidate which of the anthropometric parameters or metabolic components were most strongly associated with psychiatric disorders.

**Methods:**

The study included 88 obese and overweight children with a body mass index (BMI) greater than 85^th^ percentile. The patients were evaluated for psychiatric disorders by a single child and adolescent psychiatrist. Forty patients diagnosed with psychiatric disorders and 48 patients with normal psychiatric evaluation were compared in terms of anthropometric and metabolic parameters. BMI, BMI-standard deviation score and BMI percentile, waist circumference, waist to hip ratio, blood pressure and pubertal stage of all patients were recorded. Fasting serum glucose, insulin, lipid profile and homeostatic model assessments of insulin resistance (HOMA-IR) were measured to evaluate the metabolic parameters. Serum and 24 hour urine cortisol levels were measured.

**Results:**

HOMA-IR in the group with psychiatric disorders was found to be significantly higher than in the group without psychiatric disorders (6.59±3.36 vs 5.21±2.67; p=0.035). Other anthropometric measurements and metabolic parameters were not significantly different between the two groups.

**Conclusion:**

An understanding of the relationships between obesity related medical comorbidities and psychiatric pathologies is important to encourage patients and their families to make successful healthy lifestyle changes and for weight management in terms of appropriate treatment.

**What is already known on this topic?**

Childhood adiposity has been reported to be related to mental health conditions such as depression, behavioral and emotional disorder, anxiety and mood disorder. BeLue and colleagues reported that adolescents who were obese were 1.6 times more likely to have depression or anxiety. Other research has demonstrated obese children are 3.1 times more likely to have anxiety symptoms and 3.6 times more likely to have depressive symptoms compared to their same-age peers.

**What this study adds?**

Our findings suggest that insulin resistance, rather than obesity-related metabolic comorbidities, is more predictive of psychiatric illness. The results of our study underline the importance of assessing psychiatric functioning in obese children, particularly those with insulin resistance. We recommend routine screening of these children for the identification of psychiatric disorders.

## Introduction

Childhood obesity is an important public health problem worldwide. The prevalence of obesity in children has risen dramatically in recent years. Childhood obesity has various and considerable adverse consequences for health outcomes (1).

There is an increasing recognition of the relationship between mental illness and obesity. Childhood overweight and obesity are reported to be more strongly associated with psychiatric comorbidities as compared to their healthy-weight peers, including those with a lower health-related quality of life, lower self-esteem and body image concerns (2,3). It is known that overweight and obese children are exposed to some difficulties in social life, such as pervasive peer victimization, weight-related teasing, weight stigma and bullying (4,5). These may complicate their physical and medical health outcomes (1,6).

Obesity related psychiatric comorbidities include a variety of psychiatric illness (2). In a study by Britz et al (7), more than 40% of the obese adolescents in their sample met Diagnostic and Statistical Manual of Mental Disorders, 4^th^ edition DSM-4 criteria for a psychiatric illness. Increased lifetime rates for low mood (42.6%), anxiety (40.4%), substance use (36.2%), somatoform (14.9%) and eating (17.0%) disorders were reported in the obese group as compared with the general population. In a large population study sample from the United States, 43297 children aged between 10-17 years were evaluated; 15% of them were overweight and 16% were obese. In this study obese children compared with children classified as of normal weight were more likely to have internalizing and externalizing problems. Attention deficit and hyperactivity disorder (ADHD), conduct disorder, depression, learning disability and developmental delay were found to be more common in obese children (8).

It is still not clear whether psychiatric disorders and psychological problems are causes or consequences of childhood obesity or whether common factors promote both obesity and psychiatric disturbances in children and adolescents. The first aim of this study was to investigate psychiatric consequences of obesity and to evaluate the associations between childhood obesity related comorbidities and psychiatric disorders in children. The second aim was to identify which of the anthropometric or metabolic parameters related to obesity had an effect on mental health. To this end, we compared obese children with and without mental disorders to reveal differences in anthropometric and metabolic parameters.

## Methods

This study was conducted in Manisa Celal Bayar University Pediatric Endocrinology and Child Psychiatry Clinics. A total of 88 obese and overweight children with a body mass index (BMI) value greater than the 85^th^ percentile for age and sex, according to growth charts from the Center for Disease Control and Prevention (CDC-2000), aged nine to 17 years, who attended or were referred to our pediatric endocrinology outpatient clinic for evaluation of obesity and related comorbidities, were included in the study. We excluded individuals with developmental delays, chronic diseases, a history of drug use, a previous diagnosis of psychiatric disorders, those with any disease affecting the endocrine system (for example hypothyroidism or Cushing’s disease) or suspected syndromes associated with obesity such as Prader-Willi and Laurence-Moon-Biedl syndromes.

The study was approved by the Local Ethics Committee of Celal Bayar University, Faculty of Medicine in Manisa (number/date: 20478486-382/11.11.2015), and written informed consent were taken from the primary caregiver and patient, before the study.

All overweight and obese patients underwent a thorough physical examination, laboratory evaluation and psychiatric assessment. The assessments were all performed by specially trained clinical research staff.

Height was measured in all subjects by a wall-mounted stadiometer and weight by a calibrated scale. All subjects were measured with no clothing other than undergarments. BMI was calculated as weight (in kilograms) divided by square of height (in metres). BMI-standard deviation (SD) score and BMI percentiles were calculated using age and gender specific norms published by the CDC (9). Obesity was defined as a BMI ≥95^th^ percentile and overweight as a BMI ≥85^th^ for age and sex (10).

Waist circumference (WC) was measured with a non-stretchable tape to the nearest 0.1 cm, midway between the lowest rib and the highest point of the iliac crest parallel to the floor, without clothing and during expiration in a standing and relaxed position (as recommended by the World Health Organization Expert Committee, 1995). Hip circumference (HC) was measured in centimetres around the widest portion of the buttocks. Waist-to-hip ratio was calculated by dividing the WC by the HC.

Findings for pubertal development were recorded according to the classification of Tanner and Whitehouse (11). A testicular volume of ≥4 mL in males, and a breast development of stage 2 and over in females, were considered to be findings consistent with puberty.

Blood samples were taken in the morning after 10 to 12 hours of night-time fasting (water permitted) for glucose, insulin and lipids including triglycerides (TG), total cholesterol and high-density lipoprotein (HDL), low-density lipoprotein cholesterol and serum cortisol measurements. A 24 hour urine sample was collected for cortisol measurement.

Insulin resistance was evaluated according to the homeostasis model assessment-insulin resistance index. Different cut-off values for prepubertal and pubertal stages were used to determine the status of insulin resistance (prepubertal >2.5, pubertal >4.0) (12).

Oral glucose tolerance test was performed after overnight fasting for 12-14 h. After glucose drink containing 1.75 g/kg glucose to a maximum of 75 g, blood samples were obtained every 30 min for 120 min for measurement of plasma glucose and insulin.

Blood pressure was taken with the appropriate cuff, systolic blood pressure (SBP) and diastolic blood pressure (DBP) were measured twice in the supine position, after a ten minute rest, using the right arm and a calibrated sphygmomanometer and the mean of these two BP values were calculated.

According to the International Diabetes Federation (IDF), metabolic syndrome (MetS) can be diagnosed in children ten to 16 years old when the following criteria are fulfilled: a WC ≥90^th^ percentile, together with two more risk factors defined as a fasting blood glucose level ≥100 mg/dL (5.6 mmol/L), a serum TG level ≥150 mg/dL (1.7 mmol/L) or being under treatment for elevated TG, HDL cholesterol <40 mg/dL (1.03 mmol/L) or being under treatment for low HDL and with a SBP ≥130 or DBP ≥85, or being under treatment for hypertension. For children 16 years and older, adult criteria can be used. Ethnic-specific WC percentiles for the Turkish population are ≥94 cm for men, ≥80 cm for women and a sex-specific cut off level for HDL are <40 mg/dL (1.03 mmol/L) in men or <50 mg/dL (1.29 mmol/L) in women. For children younger than ten years of age, MetS cannot be diagnosed, but vigilance is recommended if WC is ≥90^th^ percentile (13).

### Psychiatric measurements included the following:

1. Kiddie Schedule for Affective Disorders and Schizophrenia for School Age Children-Present and Lifetime Version was used (14). This is a semi-structured interview developed by Kaufman et al (14), to evaluate present and lifetime psychopathology in children and adolescents according to DSM-3-R and DSM-4 criteria. The reliability and validity study of the Turkish translation was conducted by Gökler et al (15). Psychiatric evaluation of obese patients was performed by the same clinician. The individuals were classified into two groups as follows: (i) obese group with normal psychiatric evaluation; (ii) obese group with psychiatric disorder.

2. A sociodemographic form was developed by the study coordinators, and included questions on parental education and vocation, physical/mental illnesses in the family and information about the patient.

### Statistical Analysis

Statistical analysis of the study was performed using the Statistical Package for Social Sciences 15.0 program (IBM Inc., Chicago, Ill., USA). Descriptive data were presented as number ± SD, frequency and percentage values. Sociodemographic data, medical, anthropometric and physical measurements for cases with and without a psychiatric disorder were analysed using the chi-square test for categorical variables, t-test for those that were normally distributed and the Mann-Whitney U test for data that were not normally distributed.

## Results

In this study, 88 obese children and adolescents were evaluated. The mean age of the participants was 13.20±2.44 (range 9-17) years. Fifty-nine (67%) were female and 29 (33%) were male. The mean weight and height of the subjects were 73.94±16.93 kg and 155.93±11.56 cm, respectively. The number of subjects attending school was 84 (95.5%).

Psychiatric disorder was found in 40 (45.5%) of the children and five of them had multiple psychopathologies. These disorders consisted of anxiety disorders in 31 subjects (35.2%), depressive disorders in two (2.3%), oppositional defiant disorders in two (2.3%) and comorbid anxiety and depressive disorders in five subjects (5.7%).

Demographic, clinical and metabolic parameters were compared in children with and without mental disorder and the results are presented in Tables 1, 2 and 3. The group with mental disorders was not statistically different from the group without mental disorder in terms of age, sex, family history of psychiatric and chronic disorders, parents’ employment status, pubertal status, degree of obesity and MetS components. Insulin resistance was significantly higher in children diagnosed with psychiatric disorder and school attendance was found to be significantly lower (p=0.035).

## Discussion

The worldwide rates for overweight and obesity in children has increased rapidly among all age groups and both sexes over the past few decades (16). Childhood obesity is associated with several short- and long-term consequences (cardiovascular diseases, hypertension, hypercholesterolemia, insulin-resistance, type 2 diabetes, pulmonary and liver disease in addition to mental disorders) (6,17,18,19,20).

In this study, we evaluated psychiatric disorders in children who were obese and overweight, and compared the anthropometric and biochemical data in individuals with and without psychiatric impairment. We also assessed whether there is a metabolic or anthropometric difference that may be related to psychopathology among obese children and investigated the association of psychiatric disorders with the MetS, diagnosed according to the criteria of the IDF.

In our study, a psychiatric disorder was detected in 40 out of 88 obese and overweight patients (45.45%). We consider that this is a very high percentage of patients who are diagnosed during screening. Anxiety disorder was found as the most common psychiatric disorder among our study sample. According to the data obtained from a review of nine previous studies about the relationship between childhood adiposity and mental health (1), our study findings were consistent in relating childhood adiposity with mental health conditions such as depression, behavioral and emotional disorder, anxiety and mood disorder. BeLue et al (21) reported that adolescents who were obese were 1.6 times more likely to have depression or anxiety. Pervanidou et al (22) demonstrated that obese children are 3.1 times more likely to have anxiety symptoms and 3.6 times more likely to have depressive symptoms compared to same-age peers. In a study by Fox et al (23), 102 adolescents were evaluated and in the overall sample, 34% had symptoms consistent with depression and 32% symptoms of anxiety. Similar results have been found in studies from Turkey. In the study by Taner et al (24), 54 obese children were evaluated and psychopathology was detected in 50% of these children. In the study of Topçu et al (25), there were significant differences among obese and control groups in terms of the total score of state-trait anxiety inventory-C and child depression inventory. Our study results are consistent with studies conducted in both Turkey and other countries.

There are many articles evaluating the relationship between ADHD and obesity in the literature. In the study of Erermis et al (26) obese cases admitted to the endocrine clinic were evaluated and 13.3% of them were diagnosed with ADHD. Cortese et al (27) analyzed 42 studies including a total of 48,161 ADHD and 679,975 control subjects and found a significant relationship between obesity and ADHD in children. In another review, Cortese and Tessari (28) presented seven studies evaluating the prevalence of ADHD in individuals referred for obesity treatment. All these studies, except one, have confirmed significantly higher rates of ADHD in individuals with obesity compared to normal weight controls. Conversely, there were no cases diagnosed with ADHD in our study. In children, ADHD affects academic achievement and social adjustment negatively in the school setting, and is mostly recognized and diagnosed during the primary school period. In this present study, a known mental illness or drug use were among the exclusion criteria and the mean age of the children was 13.2. Thus most of them were of the secondary school age. In this age group, children have high rates of previous ADHD diagnosis and were therefore presumably excluded from this study which explains why we did not find any ADHD diagnosis in the study group.

It has been reported that when obesity is accompanied by a psychiatric disorder, the children become disoriented to obesity treatment along with a decrease in their school performance. Also, their body sense becomes more negative and their quality of life more distorted (24,29). With the diagnosis and treatment of the existing psychiatric disorder and improved self-esteem or improvement in other factors associated with mental health, obese individuals may be more successful in increasing their motivation (30). The present study emphasizes the importance of mental health assessment prior to treatment in order not to miss diagnoses that may affect the outcome of the treatment. Establishment of multidisciplinary teams and psychiatric evaluation are important in the effective treatment of obesity.

The relationship between obesity and mental disorder has not yet been clearly elucidated and questions such as which one triggers the other or *vice versa* or whether they co-occur remain to be clarified. The association of psychiatric disorders such as depression and anxiety disorders with MetS are relatively well-documented in adults (31). There are only limited data on the nature of the association between obesity related MetS and other comorbidities and psychiatric disorders in children.

A number of studies have documented the association of depressive symptoms or disorders with the MetS (32,33,34,35). It was reported that the MetS in childhood predicted higher levels of depressive symptoms in adulthood (36). The association of the MetS with anxiety disorder has received significantly less attention and the results of the studies on this issue are controversial. Some authors have reported more severe anxiety symptoms and more frequent anxiety disorders in MetS patients, while other researchers have not confirmed this association (37,38,39,40).

Phillips and Perry (31) compared depression and anxiety symptoms among metabolically healthy and unhealthy obese and non-obese individuals. The risk of anxiety and depressive symptoms were found to be greater among the metabolically unhealthy, obese subjects than the metabolically healthy, non-obese individuals. Increased risk for these conditions was not observed among the metabolically healthy obese subjects. Hamer et al (41), investigated whether the association between obesity and depressive symptoms is dependent on an individual’s metabolic health and report that the metabolically unhealthy obese had increased risk of depressive symptoms after a two year follow-up but that this relationship was not found in metabolically healthy, obese individuals. Furthermore, the data obtained from a recent analysis of eight studies by Jokela et al (42), demonstrate that obese individuals with a favorable metabolic profile have a slightly increased risk of depressive symptoms compared with non-obese, but the risk is greater when obesity is combined with an adverse metabolic profile. Phillips and Perry (31) investigated associations between the metabolic risk factors and depressive symptoms and anxiety among metabolic unhealty, obese subjects. Insulin resistance and abdominal obesity were associated with depressive symptoms, only insulin resistance remained significant in adjusted models for both depressive symptoms and anxiety.

We expected that accompanying obesity-related comorbidities or MetS components, rather than obesity alone, would relate to impaired psychiatric functioning and greater psychiatric distress. In view of the MetS and related components, we found that in our sample children with psychiatric disorders had higher measures of insulin resistance. We know that, obesity related cardiometabolic comorbidities are less common in children and tend to occur later in life. Insulin resistance, which is the most common early onset obesity-related comorbidity, is significantly associated with an increase in the frequency of mental disorders, even when other metabolic changes have not yet begun. We believe that this is an important outcome for this study.

Obesity and mental disorders share some behavioral factors and adverse dietary habits but are also related to different stress systems such as disturbances in the hypothalamic-pituitary-adrenal axis and dysregulation of the central serotonin, norepinephrine and dopamine neurotransmitter systems which may contribute to changes in body composition and metabolic parameters (43,44,45,46,47). Endocrinologic abnormalities may play a role in the association between psychiatric disorders and insulin resistance. It has been shown that fluoxetine, improves insulin-mediated glucose utilization independent of its effect on body weight (48). These findings indicate that the serotonergic system plays a role in the pathogenesis of both mental disorders and insulin resistance and may have a role linking these two pathogeneses. In the current study, our findings suggest that psychiatric disorders may affect peripheral insulin sensitivity, possibly via behavioral and/or neuroendocrinologic pathways.

Comorbid psychiatric disorders and related lifestyle factors affect the development of insulin resistance in obese patients. It may be predicted that adequate treatment of psychiatric disorders resulting in improvement of psychopathology related factors, such as an increase in daily physical activity, improvement in sleep disturbances, or changes in eating behavior will improve the insulin resistance. Thus, early diagnosis and adequate treatment of an underlying psychiatric disorder in obese children are very important for the improvement of impaired insulin sensitivity and may serve to decrease the risk of developing diabetes, hypertension and cardio-vascular disease in these subjects.

### Study Limitations

This study has some limitations. Firstly, it is unclear from this study whether obesity worsens psychosocial factors or psychosocial factors worsen obesity. There may also be other factors affecting these relationships, such as personal characteristics of the patient, duration of the psychiatric illness and family stress factors which have not been analyzed in the present study. Secondly, the severity of the mental disorders was not assessed in this study. Longitudinal data are needed to understand the nature of the relationship between obesity and mental disorders, as well as to document any changes in psychosocial functioning with reduction in BMI.

## Conclusion

In conclusion, our findings suggest that insulin resistance, rather than obesity-related metabolic comorbidities, is more predictive of psychiatric illness in younger obese patients. The results of our study underline the importance of assessing psychiatric functioning among obese children, particularly those with insulin resistance. Routine screening of these children is recommended for the identification of psychiatric disorders and the identification of patients who require clinical intervention. In the absence of such information, it is unlikely that lifestyle recommendations will be successful in weight management of obese patients. Also, screening for presence of the MetS in those with psychiatric disorder may help to reduce the risk of developing cardiovascular disease and type 2 diabetes mellitus.

## Figures and Tables

**Table 1:**
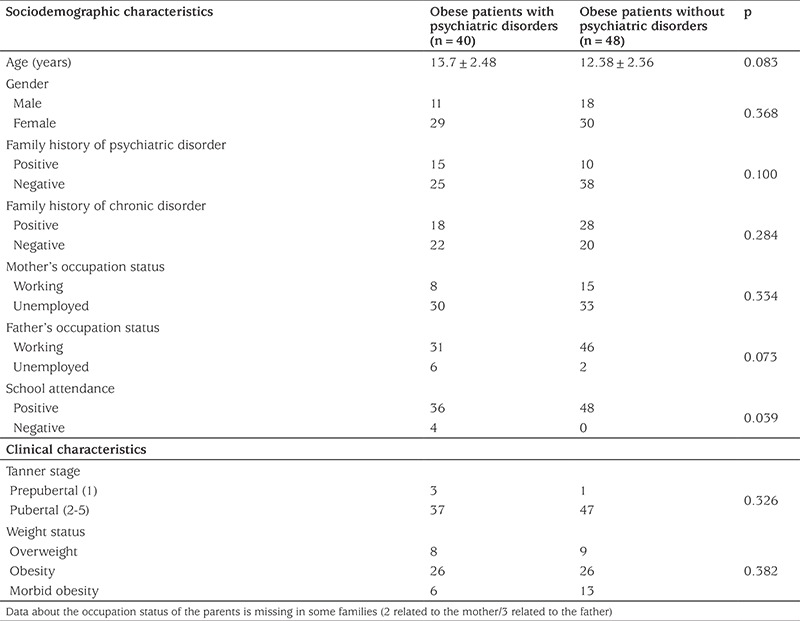
Demographic and clinical characteristics of obese patients with and without psychiatric disorders

**Table 2:**
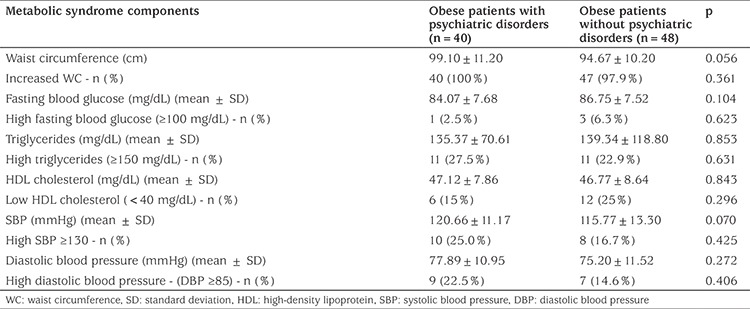
Metabolic characteristics of obese patients with and without psychiatric disorders

**Table 3:**
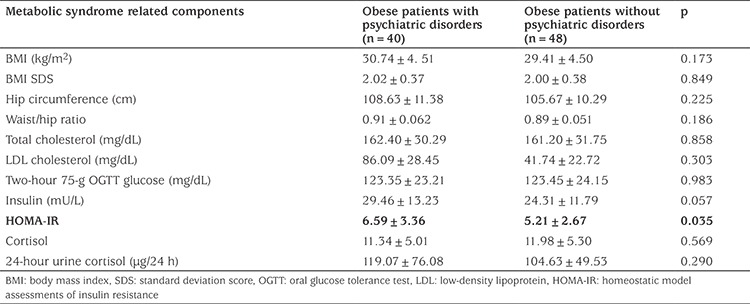
Metabolic syndrome related components in obese patients with and without psychiatric disorders (mean ± standard deviation values are given)
